# Caged Children and Christian Civilisation

**Published:** 1896-11-21

**Authors:** 


### The Hospital

A Journal of

The Medical Sciences and Hospital Administration, with

Vol. XXI.—No. 530.] AN EXTRA NURSING SUPPLEMENT. [Nov. 21, 1896.

### Caged Children and Christian Civilisation.

On the third of October we published a letter from a "Medical Correspondent," who, having recently visited the St. Mary's Home of the Kilburn Sisters, at Broadstairs, had been moved to indignation by the sight of some three hundred cubicles, each having "a strong resemblance to a cage in a travelling menagerie," and each the separate and locked-up sleeping place of an orphan child. Two things in connection with these sleeping "cages" had fastened themselves upon the consciousness of our "Medical Correspondent." One was the danger which the three hundred children were exposed to in their locked-up cages, and the other the incompatibility of such stern restraint with the ideas which now prevail as to the training and due disciplining of all classes of children. In our judgment, our medical correspondent was right in making public the facts of the case, and in expressing his opinions thereupon. After the publication of the letter, another medical gentleman, Mr. Thomas Raven, of Broadstairs, medical officer to St. Mary's Home, and a highly respected practitioner of thirty years' standing, entered the lists in defence of the cages, and of the general methods of discipline employed at St. Mary's Home. Mr. Raven wrote with some warmth, but did not, we think, succeed in making out a strong case for his clients.

This question is one for the public, and not alone for Kilburn Sisters or isolated medical men, to consider and decide upon. In considering it we must go back upon first principles. And the very first question one asks is this, Is an orphan home a mere place of detention—a prison, in fact; or is it a "home" in the proper sense of the word? The next is this, What is the aim in the training of all children who are intended to maintain themselves in after life by their own independent exertions? Is the aim, or should the aim be, to make them drilled machines, capable of working only on drilled lines at the word of command; or should it be to make them thinking, self-reliant, and trustworthy human beings of the type of human beings trained and educated under ordinary circumstances? There cannot be any doubt that if orphanages are to discharge the noble duties which the public expect them to discharge, and if they are to be such places as will satisfy a scientifically enlightened public conscience, the chief result they must aim at is to produce youths and maidens, men and women as much like ordinary good men and women as possible, and as capable of blending with, and taking part in the duties, struggles, and responsibilities of the ordinary population as possible. It is by standards such as these that the work of all orphanages and like institutions must be tried; and if the work of all orphanages and similar institutions, why not the work of the orphanages under the care and control of the Kilburn Sisters?

Let us insist upon and emphasise the contention that it is a wrong and a cruel thing so to train orphan children that they shall go through life with the mark of "orphanhood" upon them. And yet, if their training differs widely from that of all other types of normal children, the result will be, and cannot but be, that not only were they "orphans," with all that the word connotes, in their early childhood, but that they will remain "orphans" with all the disabilities and unhappiness which are involved in the fact, to the very end of their lives.

There can then hardly be any doubt that the Kilburn Sisters are making a grave and an injurious mistake in persisting, in spite of a widely-expressed public opinion to the contrary, in retaining the locked-up cages for children's cubicles during the night. Let the reader think of this one fact, and he will probably require no further thought to enable him to decide the question. The orphanages under the control of the Kilburn Sisters are the only institutions of their kind known to charitable life in which the locked-up cage system is still retained. We do not say that the Kilburn Sisters are cruel in their behaviour to the children, but we do say that the use of the locked cages is a mistaken method of dealing with them, dangerous to life, injurious to the sense of freedom, and calculated to impress upon young and imaginative minds a lifelong sense of inferiority, degradation, and humiliation which no human being outside the criminal classes should ever be subjected to.

In a sentence or two let us try to define the responsibility of subscribers and the public towards the helpless orphans who are placed under the charge of the Kilburn Sisters, and in other institutions similar to those at Kilburn and Broadstairs. Where does responsibility actually begin? It begins with the admission of each individual child to the orphanage. How long does responsibility continue? It continues during the whole period of each child's stay at the orphanage. When does responsibility end? It may never really end; but it certainly does not terminate until the child has left the institution finally, and has entered upon a separate career. In short, the question is not local, nor limited to the Kilburn Sisters. It is one which lies at the root of the right and efficient working of all orphanages and all charitable institutions. It is really this, and nothing else—Will a Christian and religious civilisation do all which intelligence, science, and conscience teach for the advantage and restoration to normal manhood and womanhood of the helpless classes which are by no fault of their own thrown on its hands, or will it compound for the performance of all the good which it knows so well how to do, but will not individually perform, by a mere money payment, leaving the actual work entirely in the hands of mere officials, whose knowledge of general principles may be hopelessly limited, or may even have no existence at all?

